# Efficacy and safety of extracorporeal membrane oxygenation combined with continuous renal replacement therapy in the management of pediatric acute respiratory distress syndrome

**DOI:** 10.3389/fped.2025.1556642

**Published:** 2025-03-18

**Authors:** Yufan Yang, Xiangni Wang, Xiulan Lu, Xinping Zhang, Jiaotian Huang, Zhenghui Xiao

**Affiliations:** ^1^ Department of Intensive Care Unit, The School of Pediatrics, Hengyang Medical School, University of South China (Hunan Children’s Hospital), Changsha, Hunan, China; ^2^ Department of Intensive Care Unit, The Affiliated Children’s Hospital of Xiangya School of Medicine, Central South University (Hunan Children’s Hospital), Changsha, Hunan, China; ^3^ Internal Medicine Teaching and Research Department, Hunan Traditional Chinese Medical College, Zhuzhou, Hunan, China

**Keywords:** acute respiratory distress syndrome, continuous renal replacement therapy, extracorporeal membrane oxygenation, pediatrics, PICU (pediatric intensive care unit)

## Abstract

**Background:**

The efficacy and safety of combining extracorporeal membrane oxygenation with continuous renal replacement therapy remain controversial. This study aimed to evaluate the efficacy and safety of extracorporeal membrane oxygenation combined with continuous renal replacement therapy in the treatment of pediatric acute respiratory distress syndrome.

**Methods:**

This retrospective study, conducted at Hunan Children's Hospital between January 2019 and December 2023, included 30 pediatric patients with acute respiratory distress syndrome who underwent extracorporeal membrane oxygenation treatment. The patients were divided into two groups based on whether continuous renal replacement therapy was used during treatment: 21 in the extracorporeal membrane oxygenation with continuous renal replacement therapy group and nine in the extracorporeal membrane oxygenation-only group. The groups were compared using *t*-test or Wilcoxon rank-sum test.

**Results:**

This study included 19 (63.3%) male and 11 (36.7%) female patients (mean age: 63.33 ± 54.41 months). The ratios of arterial partial pressure of oxygen to fraction of inspired oxygen before and at withdrawal of extracorporeal membrane oxygenation were 58.50 (40.75–70.31) and 257.00 (113.25–358.33) mmHg, respectively (*P* < 0.05). In the extracorporeal membrane oxygenation with continuous renal replacement therapy group, 21 patients (70.0%) underwent continuous renal replacement therapy, including those with acute renal injury (*n* = 5), fluid overload (*n* = 13), hyperkalemia (*n* = 3), and removal of inflammatory mediators (*n* = 3), and improvement was observed.

**Conclusions:**

The combination of extracorporeal membrane oxygenation and continuous renal replacement therapy provides safe and effective respiratory support for pediatric patients with severe acute respiratory distress syndrome and enables effective fluid-balance management, removal of inflammatory factors, and maintenance of electrolyte equilibrium.

## 1 Introduction

Acute respiratory distress syndrome (1) (ARDS) is an acute, diffuse, inflammatory lung injury with a mortality rate as high as 58% and a higher mortality rate in severe cases of ARDS. When other treatment strategies for ARDS fail, the use of extracorporeal membrane oxygenation (ECMO) is recommended for patients with severe ARDS (2–4). However, studies have identified several complications during ECMO therapy, including fluid overload, acute kidney injury (AKI), elevated inflammatory markers, and electrolyte imbalances. These complications can increase the mortality rate among patients with ARDS undergoing ECMO (5). To address these complications, several studies have proposed combining continuous renal replacement therapy (CRRT) with ECMO to improve survival rates in pediatric patients (6, 7).

The efficacy and safety of combining ECMO with CRRT in children remain controversial. International research on ECMO for ARDS is limited and predominantly focuses on adults. In contrast, the development of ECMO technology for children is still in its early stages in China, with few studies on the clinical efficacy of combining ECMO with CRRT for pediatric ARDS. This study evaluated the efficacy and safety of combining ECMO with CRRT in the treatment of pediatric ARDS.

## 2 Methods

### 2.1 Study participants

This retrospective study analyzed data from 30 pediatric patients with ARDS who received ECMO treatment at Hunan Children's Hospital between January 2019 and December 2023. Patients were grouped based on whether CRRT was required during treatment, resulting in two groups: 21 patients in the ECMO + CRRT group and nine in the ECMO group.

### 2.2 ECMO indication criteria

The criteria for initiating ECMO were (1) respiratory failure: severe respiratory failure leading to an arterial partial pressure of oxygen (PaO_2_)/fraction of inspired oxygen (FiO_2_) <60–80 mmHg or an oxygenation index >40 (in children, the recommended duration is based on adult standards: PaO_2_/FiO_2_ <60 mmHg for >3 h or PaO_2_/FiO_2_ <80 mmHg for > 6 h); (2) ineffectiveness of conventional mechanical ventilation and/or other forms of salvage therapy (such as high-frequency oscillatory ventilation, inhaled nitric oxide, or prone ventilation); (3) high ventilatory pressure parameters (such as mean airway pressure > 20–25 cm H_2_O during conventional ventilation or > 30 cm H_2_O during high-frequency ventilation or evidence of iatrogenic barotrauma); and (4) persistent, severe respiratory acidosis (such as pH <7.2) or concurrent hypoxemia despite appropriate ventilatory management (8).

### 2.3 CRRT indication criteria

Absolute indications for CRRT include refractory fluid overload despite diuretic therapy and severe hyperkalemia (> 6.5 mmol/L) or rapidly rising potassium levels with cardiac toxicity. Relative indications include (1) renal indications: when patients with AKI cannot tolerate fluid balance and metabolite fluctuations and (2) non-renal indications: persistent fluid overload (Defined as Accumulated fluid balance of weight increases by 10% compared to baseline weight.), severe electrolyte imbalances, or acid–base disturbances (9).

### 2.4 Data collection

The data collected included (1) general indicators: age, sex, and body mass index (BMI); (2) treatment-related indicators: duration of ECMO treatment, CRRT indications, duration of CRRT treatment, fluid balance (input and output) in patients who underwent CRRT, and changes in laboratory parameters before and after ECMO initiation and withdrawal; (3) complications: catheter-related bleeding, gastrointestinal bleeding, neurological complications, hemolysis, thrombosis, liver-function impairment, disseminated intravascular coagulation (DIC), and severe thrombocytopenia; and (4) outcome indicators: survival status after ECMO withdrawal.

### 2.5 Statistical methods

Data analysis was conducted using SPSS version 22.0. Normally distributed continuous variables are expressed as mean ± standard deviation (X¯ ± S), while non-normally distributed continuous variables are expressed as median (interquartile range) [M (Q1–Q3)]. Categorical data are described using percentages. The normally distributed continuous variables were compared between the two groups using independent sample *t*-test, while non-normally distributed variables were analyzed using Mann–Whitney *U*-test. For paired data from the same participant before and after treatment, if the differences followed a normal distribution, paired *t*-test was used and if the differences were severely skewed, Wilcoxon signed-rank test was applied. Categorical variables were analyzed using chi-squared test, with *P* < 0.05 considered statistically significant.

## 3 Results

### 3.1 Comparison of general and laboratory data

A total of 30 pediatric patients with ARDS receiving ECMO treatment were included in this study, as detailed in Table 1. Laboratory comparisons within 24 h before ECMO initiation showed no statistically significant differences. However, within 24 h after ECMO withdrawal, the serum creatinine levels in the ECMO + CRRT group were significantly higher than those in the ECMO group (*P* < 0.05), as shown in Table 2.

**Table 1:** Comparison of general data between ECMO and ECMO + CRRT groups.

Item	ECMO group	ECMO + CRRT Group	*t/Z/c* ^2^	*P*
Age (months)	28.00 (15.50–94.50)	65.00 (13.50–116.50)	−0.820	0.415
BMI (kg/m^2^)	16.59 ± 2.83	17.52 ± 3.48	−0.360	0.717
Male, *n* (%)	7 (77.78)	12 (57.14)	1.160	0.419^a^
PRISM III	16.00 (14.00–19.50)	17.00 (17.00–20.50)	−0.273	0.785
P-MODS	6.00 (4.00–9.00)	6.00 (5.00–7.00)	−0.391	0.696
P-SOFA	12.00 (10.00–14.00)	13.00 (12.00–14.00)	−1.053	0.292

BMI, body mass index; CRRT, continuous renal replacement therapy; ECMO, extracorporeal membrane oxygenation. P-MODS, PRISM III, P-SOFA.

^a^ Fisher's exact test.

**Table 2:** Comparison of laboratory data after ECMO withdrawal between ECMO and ECMO + CRRT groups.

Item	ECMO group	ECMO + CRRT Group	*t/Z*	*P*
White blood cell count (10^9^/L)	8.08 (5.15–15.45)	11.7400 (7.68–16.89)	−1.177	0.239
Hemoglobin (g/L)	104.00 (89.00–113.50)	96.00 (89.00–106.50)	−1.200	0.230
Platelets (10^9^/L)	63.00 (37.50–95.50)	79.00 (54.50–97.50)	−0.950	0.342
Total bilirubin (μmmol/L)	16.90 (9.85–83.05)	12.90 (9.75–32.40)	−0.792	0.428
Direct bilirubin (μmmol/L)	6.40 (2.70–41.55)	6.70 (3.30–12.35)	−0.068	0.946
Alanine aminotransferase (IU/L)	26.00 (10.70–56.40)	18.30 (10.00–60.80)	−0.249	0.803
Aspartate aminotransferase (IU/L)	49.40 (20.50–124.75)	60.50 (31.10–227.00)	−0.792	0.428
Albumin (g/L)	28.00 (24.75–30.60)	28.50 (26.45–33.75)	−0.838	0.402
Urea nitrogen (mmol/L)	5.86 ± 3.62	7.43 ± 3.86	−0.973	0.331
Creatinine (μmol/L)	29.30 (19.09–35.90)	45.60 (39.50–88.55)	−2.874	0.004
C-reactive protein (mg/L)	16.19 (10.14–81.33)	19.32 (8.20–52.63)	−0.113	0.910
Procalcitonin (ng/ml)	1.23 (0.39–1.74)	2.50 (0.63–8.37)	−1.426	0.154
Activated partial thromboplastin time (s)	94.80 (63.70–169.90)	88.70 (54.95–108.20)	−0.634	0.526
Prothrombin time (s)	16.80 (13.95–18.85)	15.70 (13.85–17.90)	−0.498	0.618
Fibrinogen (mg/dl)	261.00 (141.00–335.50)	192.00 (163.00–254.50)	−0.973	0.33
Thrombin Time (s)	240.00 (29.95–240.00)	139.60 (26.25–240.00)	−0.668	0.504
D-dimer (μg/ml)	10.87 (2.50–21.51)	5.03 (1.49–9.95)	−1.109	0.268

CRRT, continuous renal replacement therapy; ECMO, extracorporeal membrane oxygenation.

### 3.2 Treatment efficacy of ECMO and ECMO + CRRT

#### 3.2.1 Efficacy of ECMO treatment

**Figure 1:**
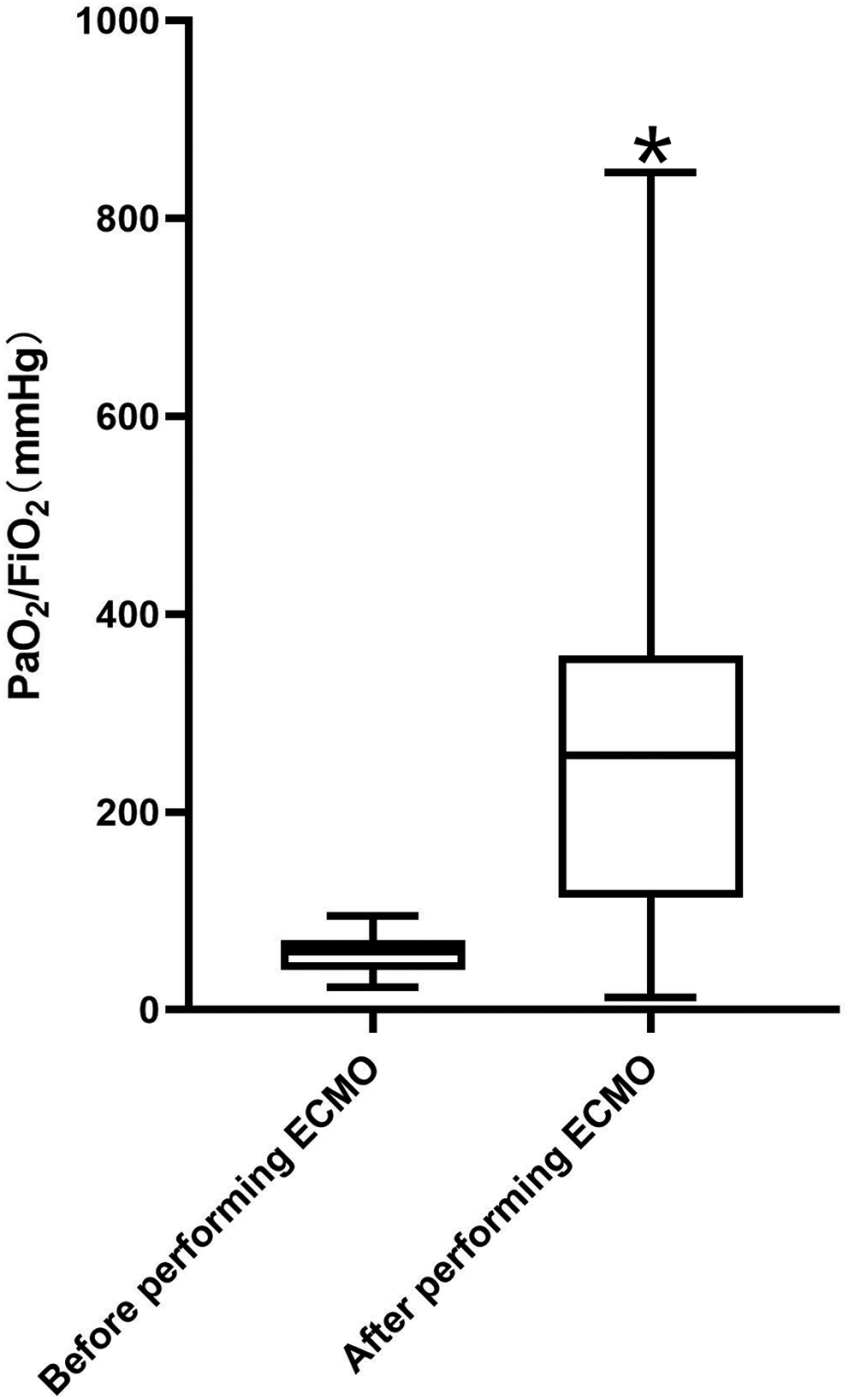
Box plot of PaO_2_/FiO_2_ before ECMO initiation and after its withdrawal. The PaO_2_/FiO_2_ ratio was 58.50 (40.75–70.31) mmHg before ECMO initiation in the patients (*n* = 30). During ECMO withdrawal, the PaO_2_/FiO_2_ ratio was 257.00 (113.25–358.33) mmHg. * represents that the difference was statistically significant (*P* < 0.05), indicating a significant improvement in the PaO_2_/FiO_2_ ratio before and after ECMO treatment. ECMO, extracorporeal membrane oxygenation; PaO_2_/FiO_2_, ratio of arterial partial pressure of oxygen to fraction of inspired oxygen.

The PaO_2_/FiO_2_ ratio was 58.50 (40.75–70.31) mmHg before ECMO initiation in the patients (*n* = 30). During ECMO withdrawal, the PaO_2_/FiO_2_ ratio was 257.00 (113.25–358.33) mmHg. This difference was statistically significant (*P* < 0.05), indicating a significant improvement in the PaO_2_/FiO_2_ ratio before and after ECMO treatment, as shown in Figure 1.

#### 3.2.2 Efficacy of ECMO + CRRT treatment

**Figure 2:**
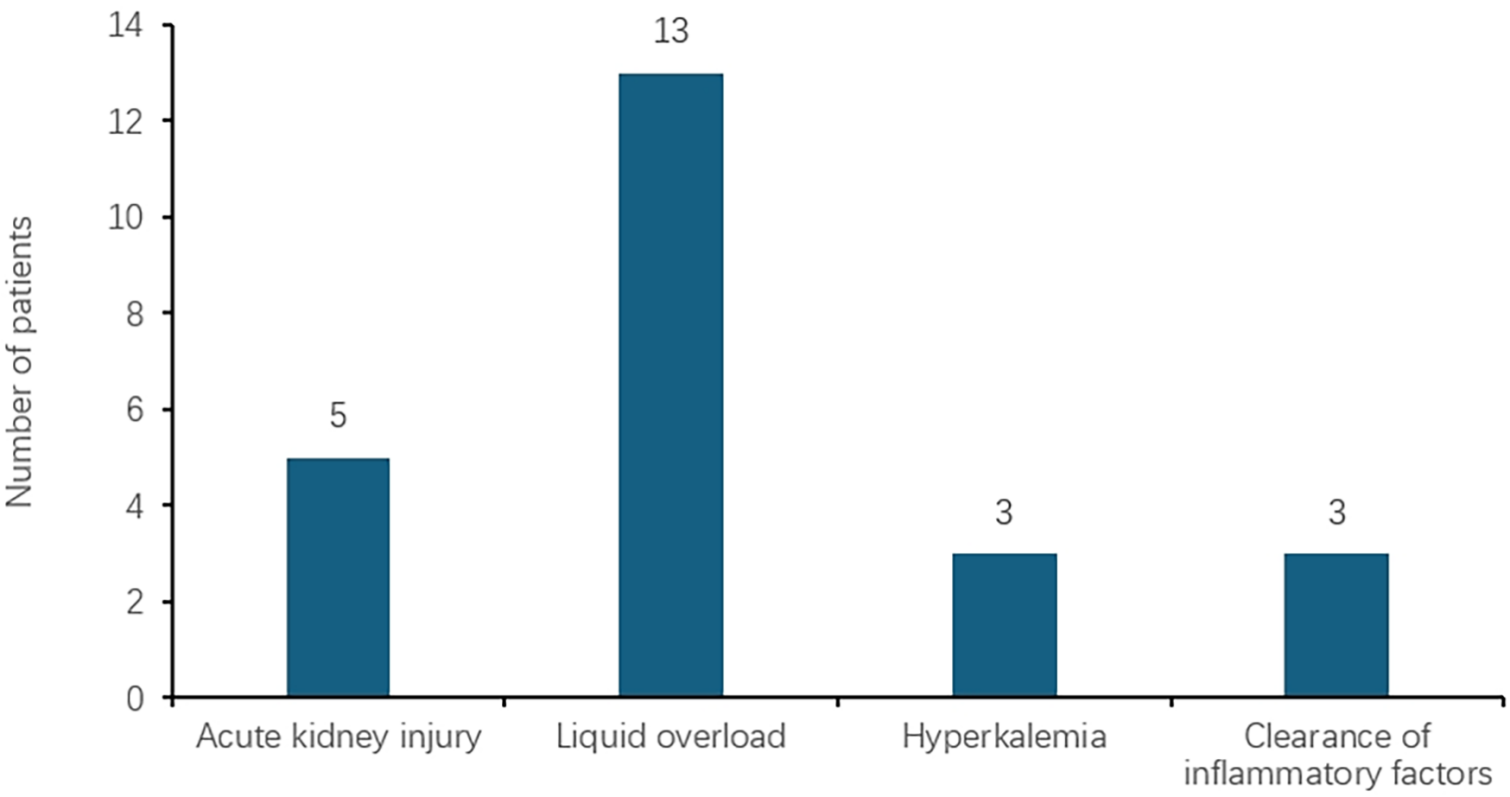
Distribution of CRRT indications among the patients in the ECMO group. Five patients underwent CRRT due to AKI, thirteen patients received CRRT to manage fluid overload, three patients underwent CRRT to address hyperkalemia. three patients received CRRT to clear inflammatory factors. CRRT, continuous renal replacement therapy; ECMO, extracorporeal membrane oxygenation.

**Figure 3:**
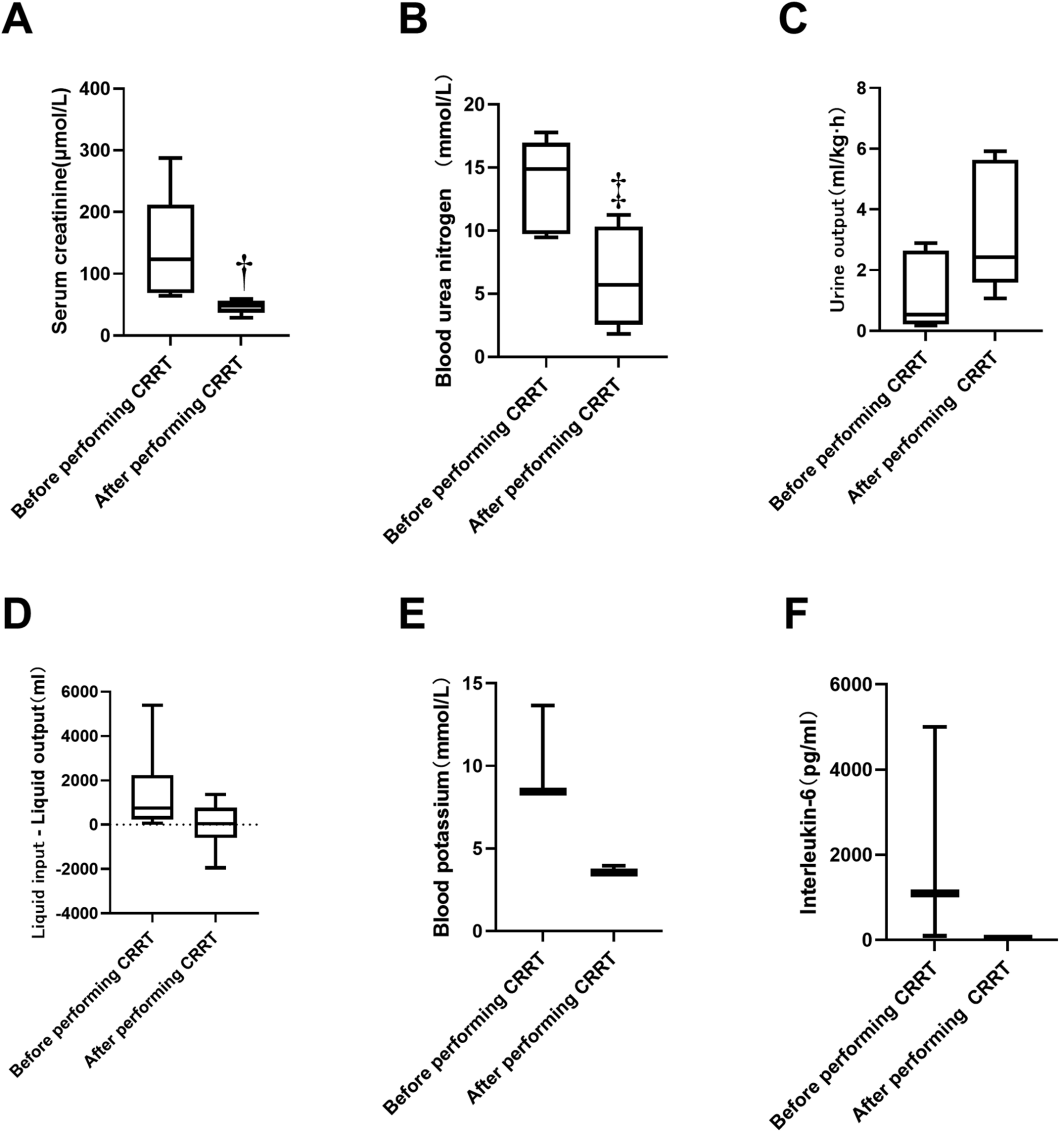
Comparison of relevant data before and after CRRT. **(A)** five patients underwent CRRT due to AKI. The serum creatinine levels before and after CRRT were 123.00 (69.15–211.60) and 48.00 (37.00–55.75) μmol/L, respectively, † reprensents a statistically significant difference (*P* < 0.05). **(B)** The blood urea nitrogen levels before and after treatment were 13.66 ± 3.73 and 6.29 ± 3.99 mmol/L, respectively, ‡ represents statistically significant difference (*P* < 0.05). **(C)** The urine output volumes before and after CRRT were 0.53 (0.22–2.63) and 2.43 (1.60–5.63) ml/(kg·h), respectively, and this difference was not statistically significant (*P* > 0.05). **(B)** The urine output volumes before and after CRRT were 0.53 (0.22–2.63) and 2.43 (1.60–5.63) ml/(kg·h), respectively, and this difference was not statistically significant (*P* > 0.05). **(D)** Thirteen patients received CRRT to manage fluid overload. **(E)** Three patients underwent CRRT to address hyperkalemia. The serum potassium levels before and after treatment were 8.45 (8.44–11.06) and 3.55 (3.53–3.75) mmol/L, respectively, with no statistically significant difference (*P* = 0.05). **(F)** Three patients received CRRT to clear inflammatory factors. The interleukin (IL) -6 levels before and after treatment were 1,095 (97.79–5,000) and 32 (9.00–69.00) pg/ml, respectively, with no statistically significant difference (*P* = 0.05). CRRT, continuous renal replacement therapy.

A total of 21 patients received ECMO combined with CRRT. Some patients had multiple indications for ECMO, as depicted in Figure 2. Among these, five patients underwent CRRT due to AKI. The serum creatinine levels before and after CRRT were 123.00 (69.15–211.60) and 48.00 (37.00–55.75) μmol/L, respectively, showing a statistically significant difference (*P* < 0.05). The distribution is shown in Figure 3A. The blood urea nitrogen levels before and after treatment were 13.66 ± 3.73 and 6.29 ± 3.99 mmol/L, respectively, with a statistically significant difference (*P* < 0.05), as illustrated in Figure 3B. The urine output volumes before and after CRRT were 0.53 (0.22–2.63) and 2.43 (1.60–5.63) ml/(kg·h), respectively, and this difference was not statistically significant (*P* > 0.05), as shown in Figure 3C. Thirteen patients received CRRT to manage fluid overload. The difference between daily fluid intake and output is depicted in Figure 3D. Three patients underwent CRRT to address hyperkalemia. The serum potassium levels before and after treatment were 8.45 (8.44–11.06) and 3.55 (3.53–3.75) mmol/L, respectively, with no statistically significant difference (*P* = 0.05), as shown in Figure 3E. Additionally, three patients received CRRT to clear inflammatory factors. The interleukin (IL) -6 levels before and after treatment were 1,095 (97.79–5,000) and 32 (9.00–69.00) pg/ml, respectively, with no statistically significant difference (*P* = 0.05), as shown in Figure 3F.

### 3.3 Supporting duration time and types of ECMO selection of two groups

The average duration of CRRT was 73.53 ± 90.86 h, and the ECMO supporting duration time in the ECMO with CRRT group was longer than that in the ECMO group 263.64 ± 201.25 h vs. 143.06 ± 123.71 h (*P* > 0.05), but there is no statistically significant difference between two groups. In the ECMO with CRRT group, there are 9 VV ECMO cases and 12 VA ECMO cases. And in the ECMO group, there are 2 VV ECMO cases and 7 VA ECMO cases. And the average rotational speed for these patients is 63.38 ± 29.70 ml*kg/min. For hemodynamically unstable patients, all patients were receiving inotropic therapy.

### 3.4 Safety of ECMO and ECMO + CRRT treatments

In the ECMO + CRRT group, 12 patients (57.1%) survived and nine (42.9%) died. In the ECMO group, three patients (33.3%) died. The difference in mortality rates between the two groups was not statistically significant (*P* > 0.05). The duration of ECMO support was longer in the ECMO + CRRT group compared to the ECMO group, but this difference was also not statistically significant (*P* > 0.05).

Complications during treatment included catheter-related bleeding, neurological complications, gastrointestinal bleeding, thrombosis, hemolysis, liver dysfunction, DIC, and severe thrombocytopenia. A comparison of these complications between the ECMO + CRRT and ECMO groups showed no statistically significant differences, as detailed in Table 3.

**Table 3:** Comparison of complications between ECMO and ECMO + CRRT groups.

Item (cases)	ECMO group	ECMO + CRRT group	χ^2^	*P*
Number of cases	9	21		
Catheter-related bleeding	2	6	0.130	>0.990
Neurological complications	0	1	0.440	>0.990
Gastrointestinal bleeding	3	6	0.068	>0.990
Thrombosis	1	6	1.074	0.393
Hemolysis	0	2	0.918	>0.99
Liver dysfunction	6	11	0.520	0.690
Disseminated intravascular coagulation	1	5	0.635	0.637
Severe thrombocytopenia	6	18	1.430	0.330

CRRT, continuous renal replacement therapy; ECMO, extracorporeal membrane oxygenation.

### 3.5 Comparative analysis of laboratory data for children receiving ECMO combined with CRRT

In the group of children who received ECMO combined with CRRT, younger children tended to be in the death group, but this difference was not statistically significant (*P* > 0.05, which is >0.05). Children with higher D-dimer values before ECMO treatment were more commonly found in the survival group, with a statistically significant difference (*P* > 0.05). After treatment, higher levels of direct bilirubin, aspartate aminotransferase (AST), and prothrombin time (PT) were associated with the death group, with statistically significant differences (*P* < 0.05). These findings are summarized in Table 4.

**Table 4:** Comparison of data at ECMO withdrawal for children receiving ECMO combined with CRRT for ARDS.

Item	Survival group	Death group	*t/z* value	*P*-value
Total bilirubin (μmmol/L)	12.00 (8.07–14.85)	17.20 (10.00–43.00)	−1.706	0.088
Direct bilirubin (μmmol/L)	3.65 (3.20–7.15)	10.7 (5.90–17.25)	−2.348	0.019
Alanine aminotransferase (IU/L)	11.80 (8.97–39.97)	41.90 (14.75–148.50)	−1.492	0.136
Aspartate aminotransferase (IU/L)	36.25 (27.53–63.95)	123.00 (83.2–625.45)	−2.559	0.010
Blood urea nitrogen (mmol/L)	6.26 ± 3.31	8.99 ± 4.16	−1.279	0.201
Creatinine (μmol/L)	45.10 (33.87–80.00)	50.00 (41.95–95.00)	−0.640	0.522
D-dimer (μg/ml)	5.19 (2.44–19.23)	2.14 (0.85–9.14)	−0.924	0.356
C-reactive protein (mg/L)	16.86 (7.02–36.48)	36.96 (9.67–82.07)	−1.563	0.118
Procalcitonin (ng/ml)	2.66 (0.53–13.54)	2.16 (0.74–4.69)	−0.569	0.570

ARDS, acute respiratory distress syndrome; CRRT, continuous renal replacement therapy; ECMO, extracorporeal membrane oxygenation.

## 4 Discussion

### 4.1 Current status of ECMO and combined Use of CRRT in treating pediatric diseases

Studies have recommended the use of ECMO in patients with severe ARDS (10). The hemodynamic changes during ECMO can lead to ischemia/reperfusion injury and may induce various complications, including systemic inflammation, hemolysis, microcirculatory dysfunction, platelet/coagulation abnormalities, fluid overload, and AKI. To address these complications, several studies have proposed the use of CRRT technology in ECMO circuits (6, 7).

Research using neonatal piglets to simulate neonatal and pediatric conditions has suggested that CRRT could help manage exacerbated inflammation, potentially facilitating repair of the alveolar capillary membrane and improving the cure rate (11). Additionally, laboratory studies (12) involving randomized groups of adult dogs have shown that CRRT can remove excess fluid from the alveoli and interstitium of ARDS-affected lungs through ultrafiltration, thereby reducing interstitial edema, enhancing lung ventilation and oxygenation. Furthermore, CRRT might remove inflammatory mediators such as tumor necrosis factor-α and IL-6 via convection and adsorption mechanisms (12), which could lower the levels of inflammatory cytokines during the initial stages of ECMO treatment after hemorrhage reperfusion and reduce lung injury while maintaining cardiac output and oxygen utilization (13), ultimately aiming to reduce mortality rates.

### 4.2 Efficacy of ECMO combined with CRRT in treating pediatric ARDS

In our center, we observed 30 children with ARDS treated with ECMO. The mortality rate was higher among those who required CRRT due to disease progression compared to those who did not require CRRT, but this difference was not statistically significant. This should not lead to the conclusion that adding CRRT increases mortality as the need for CRRT arises from disease changes during treatment. The mortality rate is influenced by the severity of the primary disease and whether the condition can be controlled during treatment.

Arterial blood gas analysis showed a significant improvement in the PaO_2_/FiO_2_ ratio before and after ECMO initiation, indicating that ECMO has a marked effect on respiratory support. However, 70% of patients with ARDS required CRRT during treatment, a proportion higher than that reported in studies of ECMO combined with CRRT for adult ARDS (14).

Regarding treatment duration, the ECMO + CRRT group had a median ECMO support duration of 263.64 ± 201.25 h, which was longer than 143.06 ± 123.71 h in the group not receiving CRRT, although the difference was not statistically significant. This finding is consistent with the results reported by Chen et al. (15).

In our study, the indications for CRRT in children receiving ECMO included fluid overload, AKI, hyperkalemia, and removal of inflammatory factors. These indications align with those identified in other studies (13). In terms of fluid management, research has shown that managing and/or preventing fluid overload is a primary indication for CRRT (16). Additionally, some studies (17) have reported that survivors of ECMO treatment had relatively lower peak fluid overload, highlighting the importance of closely monitoring fluid balance. Maintaining a negative fluid balance is crucial for the effective treatment of ARDS.

In this study, a notable imbalance was observed in fluid intake and output before CRRT initiation, which approached equilibrium after CRRT was withdrawn, suggesting that CRRT has a certain therapeutic effect on fluid overload. Serum creatinine concentration and urine output are the two classic standards for diagnosing AKI. In our study, a decrease in blood creatinine and blood urea nitrogen levels and an increase in urine output were observed post-treatment, consistent with the findings by Murphy et al. (18), indicating an improvement in renal function following CRRT treatment. Three patients received CRRT due to hyperkalemia, with a marked decrease in serum potassium levels after CRRT. Although the difference was not statistically significant, this may be attributed to the small sample size. Regarding inflammatory markers, some studies have suggested that CRRT can mitigate inflammatory responses during ECMO treatment (19, 20), potentially reducing the levels of inflammatory cytokines during the initial phase of ECMO treatment following reperfusion injury while maintaining cardiac output and oxygen utilization. In our study, three patients underwent CRRT due to significantly elevated levels of inflammatory factors. Although IL-6 levels decreased significantly between before and after CRRT, the difference was not statistically significant and might be related to the small sample size in our study.

### 4.3 Safety of ECMO combined with CRRT in the treatment of ARDS in children

Neurological complications can significantly impact patient mortality and long-term quality of life. Previous studies report an incidence of neurological complications in patients who underwent ECMO ranging from 18.4% to 36% (21, 22). In this study, one patient experienced neurological complications during treatment. These complications may be related to factors such as hypoxia and hypotension before ECMO initiation, ischemia reperfusion, and hemodynamic instability during the treatment (23–25).

Hemolysis is a potential complication associated with the combination of ECMO and CRRT (26). In our study, the incidence of hemolysis was 6%. On comparison of the ECMO + CRRT group with the ECMO-only group, the difference was not statistically significant. Hemolysis can be caused by multiple factors, including shear stress, positive pressure, and wall impact during treatment. Higher pump speeds and pressures in ECMO circuits have been linked to an increased risk of hemolysis (27). Therefore, selection of appropriate pump settings is important to balance the risk of hemolysis with survival rates.

Thrombocytopenia is common in patients who undergo ECMO, with our center observing a severe thrombocytopenia rate of 80%. Achieving the optimal balance between preventing bleeding and thrombosis in patients who undergo ECMO remains a challenge. Some suggest that the addition of CRRT might increase blood turbulence, potentially exacerbating platelet reduction (28). Therefore, regular monitoring of blood counts and coagulation profiles is essential during treatment. Collection of extensive data is crucial to identifying key factors for balancing anticoagulation and hemostasis in the future, aiming to address this challenge effectively.

### 4.4 Risk factors for mortality in children with ARDS treated with ECMO combined with CRRT

In this study, a significant decrease in AST and alanine aminotransferase (ALT) levels between before and after ECMO treatment was more common in the survival group, while those who did not experience significant improvement were more likely to be in the death group. The difference between the survival and death groups was statistically significant. Ortiz et al. (29) reported that more than half of the cases showed elevated AST and ALT at the start of ECMO treatment, but liver function typically improved after a few days. Therefore, controlling liver-function markers such as transaminases and bilirubin during treatment is crucial for physicians.

Intracranial hemorrhage (ICH) has a high mortality rate, ranging from 32% to 100% (30, 31). Due to the high mortality associated with ICH, it was included in the neurological complications category for statistical analysis. Our study found that neurological complications were significantly associated with mortality, with a significant difference observed between the survival and death groups. ICH may be related to various factors, and its high mortality rate underscores the need for prevention and close monitoring of ICH occurrence during treatment. Additionally, the use of analgesics and sedatives in patients who undergo ECMO can affect neurological assessments and may delay the detection of brain injuries caused by ECMO. Recent attention has turned to bedside brain monitoring (32), which is valuable for the early and rapid detection of brain injury in patients undergoing ECMO.

### 4.5 Limitations of the study

First, this study was a single-center, retrospective observational study with a limited sample size. Individual differences may influence the final statistical results. Future research should involve a larger sample size and multi-center randomized controlled trials to validate the findings. Second, this study did not include long-term follow-up of discharged patients. Long-term follow-up could provide more confirmatory results regarding the outcomes and effectiveness of the treatment.

In conclusion, the use of ECMO combined with CRRT in treating severe ARDS in children provides effective and safe respiratory support. This approach facilitates efficient fluid-balance management, removal of inflammatory factors, and maintenance of electrolyte homeostasis, making it a safe and effective treatment option.

## Data Availability

The original contributions presented in the study are included in the article/Supplementary Material, further inquiries can be directed to the corresponding authors.
